# Long-distance communication and signal amplification in systemic acquired resistance

**DOI:** 10.3389/fpls.2013.00030

**Published:** 2013-02-22

**Authors:** Jyoti Shah, Jürgen Zeier

**Affiliations:** ^1^Department of Biological Sciences, University of North TexasDenton, TX, USA; ^2^Department of Biology, Heinrich-Heine-UniversityDüsseldorf, Germany

**Keywords:** azelaic acid, dehydroabietinal, glycerol-3-phosphate, methyl salicylate, pipecolic acid, DIR1

## Abstract

Systemic acquired resistance (SAR) is an inducible defense mechanism in plants that confers enhanced resistance against a variety of pathogens. SAR is activated in the uninfected systemic (distal) organs in response to a prior (primary) infection elsewhere in the plant. SAR is associated with the activation of salicylic acid (SA) signaling and the priming of defense responses for robust activation in response to subsequent infections. The activation of SAR requires communication by the primary infected tissues with the distal organs. The vasculature functions as a conduit for the translocation of factors that facilitate long-distance intra-plant communication. In recent years, several metabolites putatively involved in long-distance signaling have been identified. These include the methyl ester of SA (MeSA), the abietane diterpenoid dehydroabietinal (DA), the dicarboxylic acid azelaic acid (AzA), and a glycerol-3-phosphate (G3P)-dependent factor. Long-distance signaling by some of these metabolites also requires the lipid-transfer protein DIR1 (DEFECTIVE IN INDUCED RESISTANCE 1). The relative contribution of these factors in long-distance signaling is likely influenced by environmental conditions, for example light. In the systemic leaves, the *AGD2-LIKE DEFENSE RESPONSE PROTEIN1* (*ALD1*)-dependent production of the lysine catabolite pipecolic acid (Pip), *FLAVIN-DEPENDENT MONOOXYGENASE1* (*FMO1*) signaling, as well as SA synthesis and downstream signaling are required for the activation of SAR. This review summarizes the involvement and interaction between long-distance SAR signals and details the recently discovered role of Pip in defense amplification and priming that allows plants to acquire immunity at the systemic level. Recent advances in SA signaling and perception are also highlighted.

## Introduction

**Figure 1:**
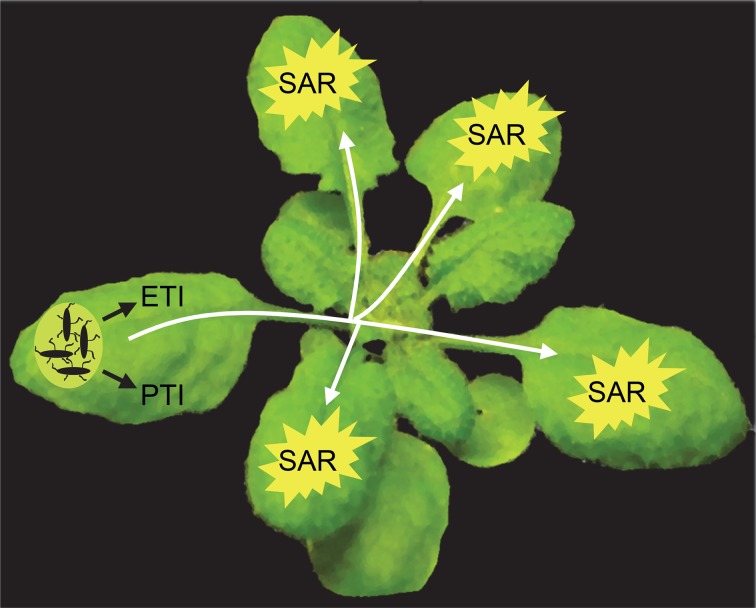
**Systemic acquired resistance.** Pathogen infection results in the activation of defenses, for example PAMP-triggered immunity (PTI) and effector-triggered immunity (ETI), in the pathogen-infected organ. Simultaneously, the infected organ releases signals that are transported to rest of the foliage, where it induces systemic acquired resistance (SAR), which protects these organs against subsequent infections by a broad-spectrum of pathogens. The phloem is a likely conduit for the transport of these long-distance SAR signals. In the distal organs, effective signal amplification must take place to guarantee SAR establishment.

Plants employ multiple layers of defense to combat pathogens. These defenses include a combination of preformed and inducible mechanisms (Jones and Dangl, 2006; Spoel and Dong, 2012). In the pathogen-inoculated tissues, recognition by the plant of molecular patterns that are conserved amongst groups of microbes results in the activation of PTI (PAMP-triggered immunity), which contributes to basal resistance that controls the extent of pathogen growth. By contrast to PTI, ETI (effector-triggered immunity), which is activated in response to plant recognition of race-specific effectors released by a pathogen, has a more pronounced impact on curtailing pathogen growth. Local infection by a pathogen can further result in immunization of the rest of the foliage against subsequent infections, a phenomenon that was reported as early as in the 1930s (Chester, 1933) and phrased “systemic acquired resistance (SAR)” by Ross (1966) (Figure 1). SAR confers enhanced resistance against a broad-spectrum of foliar pathogens. The beneficial effect of SAR has also been suggested to extend to the roots (Gessler and Kuc, 1982; Tahiri-Alaoui et al., 1993). The protective effect of SAR can be transferred to the progeny (Luna et al., 2012) and can confer a fitness advantage under conditions of high disease pressure (Traw et al., 2007).

Resistance in foliar tissues can also be enhanced by mycorrhizal associations and colonization of the rhizosphere by biocontrol fungi (Liu et al., 2007; Shoresh et al., 2010). Similarly, root colonization by plant growth-promoting rhizobacteria also enhances disease resistance in the foliage, a phenomenon that has been termed “induced systemic resistance (ISR)” (van Loon, 2007). SAR and ISR engage different mechanisms and as a result have an additive effect on foliar disease resistance (van Wees et al., 2000). SAR results in a heightened state of preparedness in the uninfected organs against subsequent infections. Furthermore, these tissues are primed to turn on defenses faster and stronger when challenged by pathogen (Conrath, 2011). Long-distance communication by the primary pathogen-infected organ with rest of the pathogen-free foliage is critical for the activation of SAR. Experiments by Joseph Kuc and colleagues led to the suggestion that this long-distance communication requires an intact phloem. In a series of grafting studies, they showed that the SAR signal can be transmitted from the pathogen-inoculated rootstock to the pathogen-free graft (scion) (Jenns and Kuc, 1979; Tuzun and Kuc, 1985). Furthermore, long-distance transmission of the SAR signal in tobacco was disrupted when the phloem tissue in the stem above the pathogen-inoculated site was removed (Tuzun and Kuc, 1985). Similarly, girdling the petiole of the primary pathogen-inoculated leaf in cucumber (*Cucumis sativus*) prevented SAR from being activated in the distal leaves (Guedes et al., 1980). In *Arabidopsis thaliana*, the SAR-inducing activity can be recovered in the phloem sap-enriched petiole exudates (Pexs) obtained from leaves inoculated with a SAR-inducing pathogen (Maldonado et al., 2002; Chaturvedi et al., 2008; Jung et al., 2009), further suggesting that the phloem is a likely conduit for transmission of the long-distance SAR signal. It has been suggested, however, that the phloem may not be the exclusive conduit for transport of the long-distance SAR signal, since defenses were also induced in distal tissues that were not connected by the path of photoassimilate translocation from the primary-infected organ (Kiefer and Slusarenko, 2003). Pexs collected from pathogen-inoculated leaves of Arabidopsis are effective in inducing SAR in tomato (*Solanum lycopersicum*), tobacco (*Nicotiana tabacum*), and wheat (*Triticum aestivum*) (Chaturvedi et al., 2008, 2012). Similarly, the SAR signal generated in the pathogen-inoculated cucumber rootstocks was found to confer protection on watermelon (*Citrullus lanatus*), and muskmelon (*Cucumis melo*) grafts (Jenns and Kuc, 1979), thus suggesting that the SAR signal is not genus- or species-specific.

## Involvement of salicylic acid signaling in SAR

SAR is accompanied by an increase in levels of salicylic acid (SA) and its derivative SA-glucoside (SAG), and elevated expression of SA-responsive genes in the pathogen-free organs. Elevated expression of the SA-responsive *PR1* (*PATHOGENESIS-RELATED 1*) gene has routinely been used as a molecular marker of SAR. SA accumulation and signaling in these organs are primed to further increase to higher levels upon challenge with a pathogen (Jung et al., 2009; Návarová et al., 2012). Genetic studies in Arabidopsis and tobacco have confirmed that SA accumulation and signaling are critical for the disease resistance conferred by SAR. The Arabidopsis *ics1* mutant, which is deficient in isochorismate synthase 1 activity that is required for SA synthesis, is SAR deficient (Wildermuth et al., 2001; Mishina and Zeier, 2007; Chaturvedi et al., 2008, 2012; Jung et al., 2009). Similarly, SAR is compromised in transgenic Arabidopsis and tobacco plants that express the SA degrading salicylate hydroxylase encoded by the *Pseudomonas putida nahG* gene (Vernooij et al., 1994; Lawton et al., 1995). In Arabidopsis, the *FMO1* (*FLAVIN-DEPENDENT MONOOXYGENASE1*) gene is required for the systemic accumulation of SA that accompanies SAR (Mishina and Zeier, 2006; Chaturvedi et al., 2012). The role of *FMO1* in SAR is discussed later in this review. The activation of SAR requires the *NPR1* (*NON-EXPRESSER OF PR GENES1*) gene, which is an important regulator of SA signaling (Durrant and Dong, 2004; Chaturvedi and Shah, 2007). NPR1 is a transcription activator that is suggested to be one of the receptors for SA (Wu et al., 2012).

SA was found to accumulate at elevated levels in phloem sap collected from cucumber and tobacco leaves inoculated with SAR-inducing pathogens (Malamy et al., 1990; Métraux et al., 1990). Hence, till the early 1990s it was thought that SA is the likely long-distance signal in SAR. However, in 1994, Vernooij and coworkers provided genetic evidence arguing against a role for SA as the long-distance signal in SAR. They demonstrated that SAR was activated in wild-type tobacco scions that were grafted onto SA-deficient NahG rootstocks, which received the primary pathogen inoculation. In contrast, SAR was not activated in NahG scions grafted on wild-type rootstocks, thus confirming that although SA is required for the disease resistance conferred by SAR, SA *per se* is not the long-distance signal in SAR. These experiments also suggest that *de novo* synthesis of SA in the pathogen-free leaves is required for SAR. Studies with tobacco plants that were unable to accumulate SA due to epigenetic suppression of phenylalanine ammonia-lyase expression, also argued against a role for SA as the long-distance signal in SAR (Pallas et al., 1996).

## Factors involved in long-distance SAR signaling

### DIR1, a lipid-transfer protein, is required for long-distance signaling in SAR

As noted above, the SAR inducing activity can be recovered in Pex collected from leaves inoculated with a SAR-inducing pathogen. The SAR inducing activity in Pex was sensitive to Proteinase K and Trypsin treatment (Chanda et al., 2011; Chaturvedi et al., 2012), thus suggesting the involvement of a protein(s) in the accumulation and/or systemic translocation of the SAR signal. The DIR1 (DEFECTIVE IN INDUCED RESISTANCE 1) protein, which exhibits structural similarities to the LTP2 family of lipid-transfer proteins, is a good candidate. *DIR1* is expressed in the phloem sieve elements and companion cells. Furthermore, DIR1 contains a signal peptide at its N-terminus that targets it for secretion to the cell surface (Champigny et al., 2011). Earlier, Maldonado et al. (2002) had identified *dir1* in a genetic screen for Arabidopsis mutants that were defective in SAR. Unlike the wild-type plant, localized inoculation with pathogen was unable to confer enhanced resistance in the distal leaves of the *dir1* mutant in response to challenge inoculation with a virulent pathogen. Although the *dir1* mutant was responsive to the SAR signal present in Avr Pex collected from wild-type plants, similar exudates collected from *dir1* when applied to wild-type plants were unable to enhance *PR1* expression and disease resistance in the distal leaves (Maldonado et al., 2002; Chaturvedi et al., 2008). Thus, it was suggested that DIR1 is required for the accumulation and/or systemic movement of a SAR inducing factor. DIR1's function in defense seems to be specific to SAR since PTI was not compromised in the *dir1* mutant (Maldonado et al., 2002). DIR1 homologs also have an important function in systemic enhancement of disease resistance in tobacco (Liu et al., 2011b). DIR1 contains two SH3 domains (Lascombe et al., 2008). Since, SH3 domains are known to facilitate interaction between proteins, these domains in DIR1 might facilitate interaction with other proteins.

### Long-distance signaling metabolites

**Figure 2:**
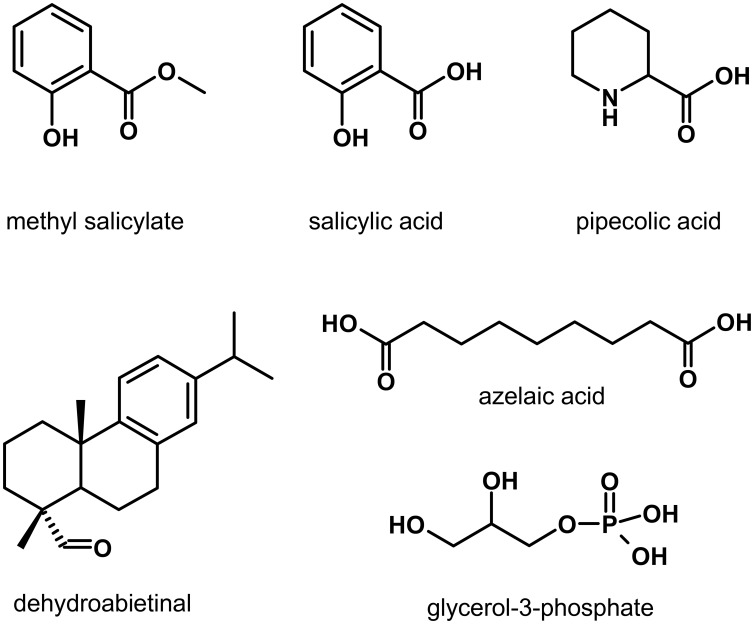
**Plant synthesized metabolites suggested to function in long-distance transport and/or signal amplification during systemic acquired resistance**.

The last 5 years have seen the identification of plant-produced metabolites (Figure 2) that are enriched in Pex after pathogen infection and/or can be systemically transported, and are thus possibly involved in long-distance signaling in SAR (Shah, 2009; Dempsey and Klessig, 2012). These metabolites can be divided into two broad groups. The first group includes methyl salicylate (MeSA) and dehydroabietinal (DA), which when locally applied promote SA accumulation in the distal leaves (Park et al., 2007; Chaturvedi et al., 2012). The second group includes azelaic acid (AzA) and pipecolic acid (Pip) that are implicated in priming the faster and stronger accumulation of SA in response to pathogen infection (Jung et al., 2009; Návarová et al., 2012). A glycerol-3-phosphate (G3P)-dependent factor has also been suggested to participate in SAR by facilitating the systemic translocation of DIR1 (Chanda et al., 2011). Evidence supporting the involvement of these molecules in long-distance communication and signal amplification in SAR is described below. Table 1 lists Arabidopsis genes/proteins involved in the synthesis and/or signaling by these metabolites.

**Table 1:** **Arabidopsis genes involved in SAR**.

**Gene**	**AtG#**	**Function**
*ALD1*	At2g13810	Aminotransferase required for pipecolic acid biosynthesis
*AZI1*	At4g12470	Putative lipid-transfer protein
*BSMT1*	At3g11480	Benzoic acid/salicylic acid methyl transferase; synthesizes MeSA
*CBP60g*	At5g26920	ACBP60 family transcription factor, involved in the control of *ICS1* expression
*DIR1*	At5g48485	Non-specific lipid-transfer protein
*FMO1*	At1g19250	Required for Pip-mediated resistance and systemic SA accumulation
*ICS1* (*SID2*)	At1g74710	Isochorismate synthase required for stress-induced SA biosynthesis
*MED15*	At1g15780	Mediator subunit 15; transcriptional co-regulator
*MED16*	At4g04920	Mediator subunit 16; transcriptional co-regulator
*MES9*	At4g37150	MeSA esterase
*MPK3*	At3g45640	MAP-kinase
*NPR1*	At1g64280	SA receptor; transcriptional coactivator
*NPR3*	At5g45110	SA receptor involved in proteasomal turnover of NPR1
*NPR4*	At4g19660	SA receptor involved in proteasomal turnover of NPR1
*PAD4*	At3g52430	Lipase-like defense regulator controlling expression of several SAR regulatory genes
*PHYA*	At1g09570	Red/far-red light perception; required for light's influence on SAR
*PHYB*	At2g18790	Red/far-red light perception; required for light's influence on SAR
*SARD1*	At1g73805	ACBP60 family transcription factor, involved in the control of *ICS1* expression
*SFD1* (*GLY1*)	At2g40690	Dihydroxyacetone phosphate reductase; synthesizes glycerol-3-phosphate in plastids

#### Methyl salicylate (MeSA)

**Figure 3:**
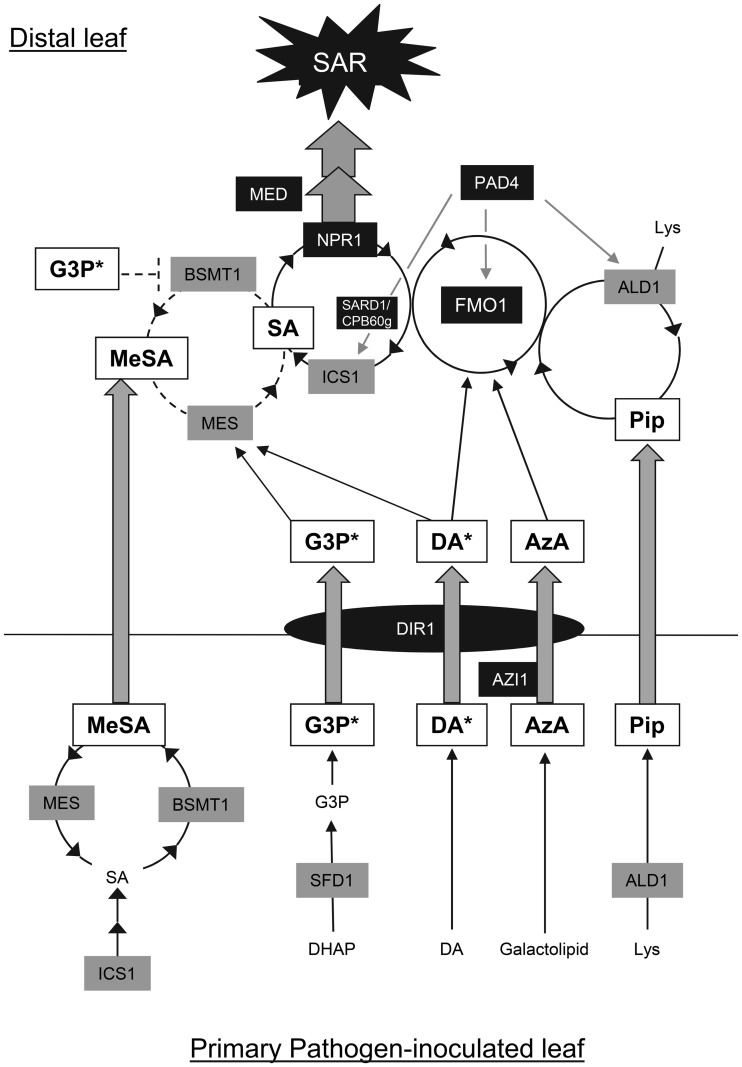
**SAR circuitry involving a network of signaling molecules.** Studies in Arabidopsis and to a lesser extent in tobacco have indicated that multiple signaling molecules participate in SAR and that the role of some of these signals is influenced by the environment. The genes listed in this model are from Arabidopsis. *Events in the primary pathogen-infected leaf:* In Arabidopsis, increased activity of ICS1, resulting from pathogen-induced expression of the corresponding gene, provokes increased SA accumulation. A fraction of the accumulating SA is converted to MeSA by BSMT1. In tobacco, the high level of SA was simultaneously shown to inhibit the MeSA esterase (MES) activity of SABP2, thus ensuring increase in MeSA level. Glycerol-3-phosphate (G3P), azelaic acid (AzA), and pipecolic acid (Pip) levels also increase in response to pathogen inoculation. SFD1 (GLY1) catalyzes the synthesis of glycerol-3-phosphate from dihydroxyacetone phosphate (DHAP). AzA has been suggested to be synthesized from galactolipids by a non-enzymatic method. Pip is synthesized from lysine (Lys) via the ALD1 aminotransferase and heavily accumulates in infected leaves. Expression of the *ALD1* gene is induced in response to pathogen inoculation. Absolute levels of DA do not change. However, DA is mobilized from a non-signaling low-molecular weight to a high molecular weight signaling DA (DA^*^) complex in response to pathogen inoculation. Trypsin treatment destroys the high molecular weight DA^*^ complex, suggesting the presence of proteins in this complex. The AzA-inducible *AZI1* gene is required for AzA-induced SAR and also promotes DA^*^-induced SAR. However, its involvement in SAR induced by the other factors is not known. DIR1, a putative non-specific lipid-transfer protein, is postulated to be involved in transport of a signal required for SAR. Genetic studies indicate that DIR1 is required for G3P, DA, and AzA-induced SAR. *Events in the distal (systemic) leaf:* Systemic transport of MeSA, a G3P-derived factor (G3P^*^), DA^*^, AzA, DIR1, and, possibly, Pip from the pathogen-inoculated leaf to the distal leaves occurs via the vasculature, most probably the phloem. G3P^*^ and DIR1 have been suggested to facilitate long-distance transport of each other. DA^*^ and G3P^*^ promote accumulation of *MES* transcript (and likely the corresponding protein). Simultaneously, G3P^*^ and DIR1 down-regulate expression of *BSMT1*, thus ensuring that the equilibrium is in favor of conversion of MeSA to SA. An amplification loop involving ALD1, Pip, FMO1, ICS1, SA, and the SA receptor NPR1, promotes Pip and SA accumulation. PAD4 regulates the expression of *ALD1, FMO1, SARD1, CPB60g*, and *ICS1*. NPR1 activation by SA leads to the expression of defense genes that contribute to SAR. MED transcriptional co-regulator subunits seem to act downstream of NPR1. Pip and FMO1 are required for the induction of *ICS1* expression and accumulation of SA in the pathogen-free distal leaves. *ICS1* expression is also controlled by SARD1 and CPB60g, a partly redundant pair of transcription factors. DA^*^, AzA and Pip signals converge at *FMO1*, which is required for activation of SAR by these signal molecules. It is likely that *FMO1* is also required for G3P^*^ and MeSA-induced SAR. However, this needs to be tested. *ALD1* is a point of convergence of the AzA and Pip pathways. Pip acting through an amplification loop involving *FMO1*, promotes *ALD1* expression and thus its own synthesis. DIR1 is essential for SAR induced by MeSA, G3P^*^, DA^*^, and AzA. Whether it is required for Pip-induced SAR is not known. DA is shown to interact synergistically with AzA and the *SFD1*-dependent mechanism. White and gray boxes represent the signaling molecules and biosynthetic enzymes, respectively. Signaling/transport proteins are represented by black boxes/ovals. Gray-filled arrows represent possible long-distance transport. Black arrows indicate positive regulation (induction), while black lines ending with a bar indicate negative regulation. The solid line used for the Pip/SA amplification cycle symbolizes a robust requirement for this part of the circuit for SAR. The contributions of MeSA, DIR1, and G3P to SAR establishment seem less prominent when plants receive a prolonged period of light after pathogen contact.

The volatile SA derivative MeSA (Figure 2), also known as the oil of winter-green, has previously been associated with plant-insect interaction and inter-plant communication (Shulaev et al., 1997; Van Poecke and Dicke, 2002; Snoeren et al., 2010). More recently, MeSA has been suggested to be involved in long-distance signaling in SAR (Dempsey and Klessig, 2012). MeSA levels were reported to increase in the *Tobacco mosaic virus* (TMV)-infected and the distal virus-free leaves of tobacco, as well as in the Pex collected from TMV-infected leaves (Park et al., 2007). TMV infection-induced SAR was attenuated in tobacco plants in which expression of the *SAMT1* (*SA-METHYLTRANSFERASE1*) gene, which encodes a MeSA synthesizing S-adenosyl-L-methionine: salicylic acid carboxyl methyl-transferase, was silenced by RNAi (Park et al., 2007). Reciprocal grafting between *SAMT1*-silenced and wild-type tobacco plants indicated that SAMT1 was required in the primary TMV-infected leaves for the induction of SAR. The MeSA esterase encoded by the tobacco *SABP2* (*SA-BINDING PROTEIN 2*) gene is also required for the activation of SAR in tobacco (Forouhar et al., 2005; Kumar et al., 2006; Park et al., 2007). A missense alteration (Ser_81_ → Ala_81_) in SABP2 that resulted in loss of its MeSA esterase activity, also resulted in the inability to restore SAR in tobacco plants lacking endogenous SABP2 activity (Park et al., 2007). Furthermore, competitive inhibition of SABP2's esterase activity by 2,2,2,2′-tetra-fluoroacetophenone, prevented the induction of SAR (Park et al., 2009). It has been suggested, as shown in Figure 3, that during the activation of SAR, SAMT1-synthesized MeSA is transported out of the pathogen-inoculated leaf to the distal leaves. In the distal leaves, MeSA is hydrolyzed by the esterase activity of SABP2 to produce SA, which along with *de novo* synthesized SA contributes to the activation of downstream signaling in the pathogen-free organs (Dempsey and Klessig, 2012).

MeSA was also shown to be required for the induction of SAR in potato (*Solanum tuberosum*) by arachidonic acid (Manosalva et al., 2010). MeSA levels increased in the arachidonic acid-treated and the distal untreated leaves of potato. Blocking MeSA accumulation by RNAi-mediated silencing of the SABP2 homolog-encoding *METHYL ESTERASE 1* (*StMES1*) gene in potato compromised arachidonic acid-induced SAR. Furthermore, as in tobacco, 2,2,2,2′-tetrafluoroacetophenone prevented the induction of SAR in potato. 2,2,2,2′-tetrafluoroacetophenone also blocked SAR in Arabidopsis (Park et al., 2009). Knock-down of expression of multiple *AtMES* genes, which encode putative MeSA esterases in Arabidopsis, also attenuated SAR, however, only in 50% of experiments (Vlot et al., 2008; Chaturvedi et al., 2012). Similarly, while Liu et al. (2010) observed that SAR was weaker in the *Arabidopsis bsmt1* mutant, which lacks a MeSA synthesizing benzoic acid/salicylic acid methyl transferase 1, Attaran et al. (2009) noted that despite the MeSA deficiency, the *bsmt1* mutant plants were SAR competent. These studies suggest that the role of MeSA in SAR in Arabidopsis is likely impacted by additional factors. Light has been suggested to be a factor that likely influences the importance of MeSA in SAR in Arabidopsis (Liu et al., 2011a). Liu et al. (2011a) noted that when the primary inoculation with the SAR inducing bacteria was conducted early during the light period, MeSA was less important for SAR. However, when the primary inoculation occurred close to the onset of the dark period, MeSA was comparatively more important for SAR.

In comparison to the wild-type plant, expression of the *BSMT1* gene and MeSA content were higher in the pathogen-inoculated and the distal leaves of the *dir1* mutant (Liu et al., 2011b). In contrast, the content of free SA and SAG were lower in *dir1* tissues. Liu et al. (2011b) have suggested that DIR1 depresses the conversion of SA to MeSA, resulting in SA accumulation in the systemic organs expressing SAR. A similar correlation between *DIR1* and *SAMT1* expression was observed in tobacco as well (Liu et al., 2011b).

#### Dehydroabietinal (DA)

Terpenoids form one of the largest families of secondary metabolites in plants (Tholl, 2006). The abietane family of diterpenoids, which are components of oleoresin produced by conifers, have pharmacological and industrial applications (Trapp and Croteau, 2001; Bohlmann and Keeling, 2008). These compounds are also produced by angiosperms (Hanson, 2009), but their function in plants is unclear. Chaturvedi et al. (2012) purified DA, an abietane type diterpenoid, as a SAR-inducing factor from Avr Pex. Deuterated DA when applied to Arabidopsis leaves was rapidly transported out of the leaf and recovered from the untreated leaves. DA is one of the most potent inducer of SAR that is active when applied as picomolar solutions to leaves of Arabidopsis, tobacco, and tomato (Chaturvedi et al., 2012). Local application of DA systemically induced SA accumulation and *PR1* expression in the untreated leaves (Chaturvedi et al., 2012). DA induced SAR was attenuated in the SA deficient NahG transgenic and *ics1 ics2* double mutant plants and in the SA signaling-deficient *npr1* mutant, thus confirming that DA functions upstream of SA accumulation and signaling. The *FMO1* gene, although not required for SA accumulation in the DA-treated leaves, was required for systemic SA accumulation in DA-treated plants and DA-induced SAR.

Unlike the other SAR signal molecules described here (Figure 2), DA content did not increase in the pathogen-inoculated leaves and Pex during SAR. However, when Avr Pex collected from Avr pathogen-treated leaves was subjected to molecular sieve chromatography, DA was found to be enriched in the biologically active HMW fraction (>100 kD) (Chaturvedi et al., 2012). By comparison, in Pex derived from mock-inoculated leaves, DA was enriched in a LMW fraction (<30 kD) that was unable to induce SAR. Chaturvedi et al. (2012) have proposed that the rate limiting step in SAR is the mobilization of DA from the biologically inactive LMW pool into a biologically active signaling form (DA^*^) that is present in the HMW pool. Trypsin treatment, which destroys the SAR inducing activity of Avr Pex, also reduced DA content in HMW, suggesting that DA is associated with proteins in the HMW pool. What are the proteins in this HMW pool? Is DIR1 one of the proteins in this pool? Additional evidence with plants that are deficient in DA^*^ are also needed to determine if DA^*^ is essential for biologically-induced SAR.

#### Azelaic acid (AzA)

In tissues exhibiting SAR, SA accumulation is primed for faster and stronger induction in response to pathogen inoculation. Azelaic acid (AzA) (Figure 2), a nine carbon dicarboxylic acid has been suggested to be a factor involved in this priming response in Arabidopsis (Jung et al., 2009). AzA levels in Avr Pex collected from Arabidopsis leaves were found to be substantially higher than in Pex collected from mock-inoculated leaves. Local application of AzA systemically enhanced disease resistance. Deuterated AzA applied to Arabidopsis leaves was recovered in Pex and in the untreated leaves, suggesting that AzA is systemically translocated through the plant. AzA-mediated resistance required SA synthesis and signaling. However, unlike MeSA and DA, AzA application was not sufficient to promote SA accumulation and *PR1* expression in Arabidopsis leaves. Instead, pathogen-induced SA accumulation and *PR1* expression were faster and stronger in plants that were previously treated with AzA, suggesting that AzA is a priming factor. *FMO1* and *DIR1* were required for AzA-induced SAR. Also required for AzA induced SAR is ALD1, an aminotransferase that is involved in the synthesis of pipecolic acid (Pip), which as described below is involved in signal amplification during SAR (Návarová et al., 2012). The *AZI1* (*AZELAIC ACID-INDUCED 1*) gene, which encodes a putative lipid-transfer protein, was transiently expressed at elevated levels in AzA-treated plants. Experiments with the *azi1* mutant confirmed that *AZI1* is required for AzA- and biologically-induced SAR. The SAR associated priming of SA accumulation/signaling were attenuated in the *azi1* mutant. Unlike Avr Pex from wild-type plants, local application of Avr Pex collected from the *azi1* mutant was unable to systemically enhance disease resistance in wild-type plants. Furthermore, while locally applied Avr Pex and AzA were capable of enhancing disease resistance in the treated leaves of wild-type and *azi1* mutant, they were unable to promote disease resistance in the distal leaves of the *azi1* mutant compared to the wild-type plant. Thus, it has been suggested that *AZI1* is required for the accumulation and/or translocation of a SAR signal (Jung et al., 2009).

A potential mechanism for the synthesis of AzA is by oxidation of 9-oxononanoic acid synthesized from fatty acids by the action of 9-lipoxygenase and hydroperoxide lyase. Indeed, mutation in the *LOX1* gene, which encodes one of the two 9-lipoxygenase in Arabidopsis, disrupts SAR (Vicente et al., 2012). However, Avr pathogen inoculation-induced accumulation of AzA was retained in the *lox1 lox5* double mutant (Zoeller et al., 2012). Zoeller et al. (2012) suggested that AzA is a general marker of lipid peroxidation that is synthesized by a free-radical based mechanism from galactolipids, rather than a general immune signal. Moreover, Návarová et al. (2012) showed that SAR can occur without the concomitant accumulation of AzA in Pex collected from virulent pathogen-treated plants. Zoeller et al. (2012) reported that AzA content in virulent pathogen-inoculated leaves was only slightly higher than in mock-inoculated leaves. This could explain the lack of AzA increase in Pex collected from virulent pathogen-inoculated leaves (Návarová et al., 2012), compared to that observed in Avr Pex (Jung et al., 2009). None-the-less, taken together these recent studies by Zoeller et al. (2012) and Návarová et al. (2012) suggest that systemic translocation of AzA is not essential for the establishment of SAR *per se*, but when it is translocated, AzA can add to the strength of systemic immunity observed during SAR.

#### SFD1-synthesized glycerol-3-phosphate-derived factor and its interplay with DIR1

*sfd1* (*suppressor of fatty acid desaturase deficiency 1*) mutants were identified in a screen for suppressors of the constitutive SAR and dwarf phenotypes of the lipid metabolism *ssi2* (*suppressor of SA-insensitivity 2*) mutant (Nandi et al., 2003, 2004), which itself was identified as a suppressor of the *npr1* mutant (Shah et al., 2001). *sfd1* mutants had defects in lipid composition, in particular levels of the plastid-localized 34:6-MGDG (monogalactosyldiacylglycerol) were lower in the *sfd1* mutant, compared to the wild-type plant, while levels of 36:6-MGDG were higher in the *sfd1* mutant. Biologically-induced SAR was compromised in the *sfd1* mutant (Nandi et al., 2004; Chaturvedi et al., 2008, 2012). The SAR defect of the *sfd1* mutant was characterized by the lack of systemic increase in SA content and *PR1* transcript in response to localized pathogen inoculation. The *sfd1* mutant was responsive to SA (Nandi et al., 2004), and local application of Avr Pex from wild-type plants complemented the SAR defect of the *sfd1* mutant (Chaturvedi et al., 2008), suggesting that the *sfd1* mutant is sensitive to the long-distance SAR signal. In contrast, Avr Pexs collected from the *sfd1* mutant were unable to induce SAR when applied to wild-type plants, indicating that the *sfd1* mutant is defective in the accumulation and/or translocation of a long-distance translocated SAR signal (Chaturvedi et al., 2008). DA content was not adversely impacted in the *sfd1* mutant. However, in agreement with a role for *SFD1* in long-distance signaling leading to systemic SA accumulation, the *sfd1* mutant exhibited reduced sensitivity to the SAR-inducing activity of DA (Chaturvedi et al., 2012).

SFD1 encodes a plastid-localized dihydroxyacetone phosphate (DHAP) reductase that synthesizes glycerol-3-phosphate (G3P) (Figure 2) (Nandi et al., 2004), an important precursor in the synthesis of several biomolecules, including membrane and storage lipids. SFD1's DHAP reductase activity and its localization to the plastids were shown to be critical for its involvement in SAR, suggesting that SFD1 synthesized G3P, or a product thereof, is required for the accumulation and/or long-distance transport of a SAR signal (Lorenc-Kukula et al., 2012). More recently, Chanda et al. (2011) showed that SAR is also attenuated in the *gly1* mutant, which contains a mutation in the *SFD1* gene in Arabidopsis accession Columbia. However, unlike *sfd1*, which is in the accession Nössen, the *gly1*allele was not defective in the SAR associated systemic enhancement of SA accumulation and *PR1* expression. In Arabidopsis, G3P levels were reported to be elevated in the pathogen-inoculated and the distal pathogen-free leaves, as well as Avr Pex (Chanda et al., 2011). Chanda et al. (2011) further showed that SAR could be restored in the *gly1* mutant by co-applying G3P with Avr Pex, thus confirming an important role for G3P, or a G3P-derived factor in long-distance signaling associated with SAR. Since locally applied ^14^C-labeled G3P could not be recovered in the systemic leaves, G3P *per se* is unlikely to be the systemically translocated SAR signal. Rather, a G3P-dependent factor is likely involved in long-distance signaling. These results also suggest that the systemic increase in G3P observed in SAR likely results from *de novo* synthesis.

Although G3P, when co-applied with Pex, was capable of enhancing disease resistance in the distal leaves, G3P by itself was not sufficient to induce systemic resistance (Chanda et al., 2011). These results suggest that additional factors that are present in Pex are required for G3P to induce SAR. An earlier study had shown that Avr Pex from *sfd1* to *dir1*, although ineffective in inducing SAR when applied individually, when co-applied were effective inducers of systemic disease resistance (Chaturvedi et al., 2008). This cross-complementation experiment suggested that the SFD1- and DIR1-dependent factors might function together in long-distance signaling. Indeed, G3P when co-applied with DIR1 protein was capable of enhancing systemic disease resistance (Chanda et al., 2011). G3P levels were also lower in Avr Pex from *dir1* mutant, leading to the suggestion that DIR1 and the G3P-dependent factor are required for systemic translocation of each other. Whether G3P or a G3P-dependent factor binds DIR1 is not known. G3P applied with Pex up-regulates *MES9* expression and simultaneously down-regulates *BSMT1* expression in the distal un-treated leaves (Chanda et al., 2011). As mentioned earlier, MES9 is a putative MeSA esterase, while BSMT1 is involved in MeSA synthesis. However, G3P application did not result in systemic increase in SA and SAG content (Chanda et al., 2011). Hence, the altered *MES9* and *BSMT1* expression may not be important for G3P-induced SAR, or alternatively their importance might be dictated by other factors. Liu et al. (2011b) showed that similar to its impact on the contribution of MeSA in SAR, light influenced the contribution of the G3P-dependent factor in SAR. The *gly1* mutant was SAR competent when the primary inoculation with the SAR-inducing microbe was conducted early during the light period. However, when the primary inoculation occurred close to the onset of the dark period, the *gly1* mutant was SAR-defective.

## SAR signaling and signal amplification in systemic leaves

Long-distance signals generated and released from the primary pathogen-inoculated leaves are supposed to be perceived by the cells in the distal organs for SAR initiation at the whole plant level (Figure 1). The receptors of individual mobile signals which activate SAR signaling in the distal organs are yet to be identified. Early signaling events result in the systemic accumulation of SA, and subsequent increases in expression of a battery of defense-related genes (SAR genes) is thought to contribute to the enhanced state of broad-spectrum resistance (Sticher et al., 1997). Compared to PTI and ETI, local forms of induced resistance that are activated upon direct pathogen contact via recognition of microbial elicitors (Jones and Dangl, 2006), induction of systemic immunity is indirectly triggered by mobile, endogenous plant signals. The overall direct defense eliciting capacity of numerous PAMPs and/or pathogen released effectors at inoculation sites is probably higher than the elicitor strength of endogenous long-distance signals in distal leaves. It has been suggested that amplification of the stimulus delivered by the SAR signals is important for SAR establishment (Mishina and Zeier, 2006). Recent findings provide evidence that pipecolic acid (Pip), a common lysine catabolite in plants and animals, acts as a central component of a feedback amplification mechanism that is critical for systemic SA accumulation and SAR (Návarová et al., 2012).

### Pipecolic acid—a critical SAR signal that orchestrates defense amplification

#### Pipecolic acid systemically accumulates in pathogen-inoculated plants

The cyclic non-protein amino acid L-Pip (homoproline; Figure 2) is present in plants throughout the plant kingdom (Morrison, 1953). L-Pip is a common catabolite of L-Lys in plants and animals (Broquist, 1991), and the pipecolate pathway represents the main degradation pathway of Lys in mammalian brains (Chang, 1976). In plants, Pip levels increase following chemical treatments that affect growth and upon osmotic stress (Yatsu and Boynton, 1959; Moulin et al., 2006). Pálfi and Dézsi (1968) reported that Pip accumulates both in virus-infected potato and tobacco and in fungus-infected rice leaves. They therefore described Pip as an indicator of abnormal protein metabolism in diseased plants. Since then, the physiological function of Pip in plants has remained elusive, albeit it was found to exert flower-inducing activity in the aquatic plant *Lemna gibba* (Fujioka et al., 1987).

Pip strongly accumulates, alongside with several other free amino acids, its precursor Lys, and another Lys catabolite, α-aminoadipic acid (Aad), in Arabidopsis leaves inoculated with SAR-inducing (virulent or Avr) *P. syringae* and in leaves treated with bacterial PAMPs (Návarová et al., 2012). Moreover, the only amino acid found to substantially increase in leaves distal from sites of pathogen inoculation in this study was Pip. Pip and SA therefore share the characteristic of systemically accumulating in plants upon localized pathogen inoculation. A time-resolved analysis in SAR-induced Arabidopsis indicates that systemic Pip levels start to significantly rise before marked elevations of SA are detectable in the systemic tissue (Návarová et al., 2012).

Pip biosynthesis and accumulation proceeds via ALD1, because the *ald1* mutant completely lacks local and systemic accumulation of Pip upon Avr or virulent *P. syringae*-inoculation (Návarová et al., 2012). *ALD1* transcript levels rise both locally and systemically in pathogen-inoculated Arabidopsis (Song et al., 2004a). *In vitro*, recombinant ALD1 has aminotransferase activity with strong substrate preference for Lys (Song et al., 2004b). It is conceivable that ε-amino-α-ketocaproic acid and Δ1-piperideine-2-carboxylic acid are direct reaction products of an ALD1-catalysed Lys aminotransferase reaction. However, the exact biochemistry of ALD1-mediated Pip production and the existence of a yet to postulate reductase that converts Lys transamination products to Pip remains to be clarified (Návarová et al., 2012).

#### The Pip resistance pathway is central for SAR

Pipecolate-deficient *ald1* plants fail to accumulate SA in distal leaf tissue following pathogen-inoculation and are fully compromised in SAR (Song et al., 2004a; Jing et al., 2011; Návarová et al., 2012). However, *ald1* plants regain the ability for systemic SA accumulation and SAR establishment when Pip is exogenously applied to the whole plant prior to pathogen treatment, demonstrating that Pip accumulation is critical for systemic SA production and SAR (Návarová et al., 2012). The *ald1* mutant also exhibits attenuated local resistance to compatible and incompatible *P. syringae*, and this is accompanied with reduced local defense responses such as SA biosynthesis, camalexin accumulation, and defense-related gene expression (Song et al., 2004a,b; Návarová et al., 2012). Exogenously applied Pip fully overrides the defects of *ald1* in PTI and ETI and increases the resistance of wild-type plants to bacterial infection. Moreover, Pip feeding of plants prior to inoculation boosts pathogen-triggered induction of SA biosynthesis, camalexin accumulation, and defense-related gene expression in wild-type and *ald1* plants, indicating that Pip strongly amplifies pathogen-triggered defense responses. The positive regulatory role of Pip on SA biosynthesis is particularly important for SA accumulation in distal leaves. It has been suggested that the early systemic increase of Pip at the onset of SAR functions as an initial trigger for signal amplification leading to the systemic increase in SA (Návarová et al., 2012).

Concomitant with SAR, localized *P. syringae* inoculation triggers enhanced expression of several hundred genes in the distal leaves of Arabidopsis wild-type plants. This massive switch in gene expression at the systemic plant level is totally lost in the *fmo1* mutant (Mishina and Zeier, 2006). The flavin-dependent monooxygenase FMO1 was previously identified as a critical regulator of SAR and found necessary for effective local resistance to several bacterial and oomycete pathogens (Bartsch et al., 2006; Koch et al., 2006; Mishina and Zeier, 2006; Jing et al., 2011). Like *ALD1, FMO1* is necessary for the systemic accumulation of SA upon SAR induction (Mishina and Zeier, 2006). In contrast to *ald1*, however, *fmo1* fails to establish Pip-induced resistance to bacterial infection. These data indicate that FMO1 functions downstream of Pip and upstream of SA in SAR (Návarová et al., 2012). Importantly, Pip enhances both its own biosynthesis and downstream signaling in SAR via amplification of pathogen-triggered *ALD1* and *FMO1* expression, indicating the existence of a positive feedback amplification loop with Pip as a central player (Figure 3; Návarová et al., 2012).

Biochemically characterized flavin-dependent monooxygenases from plants, animals, or fungi oxidize either N- or S-containing functional groups within small metabolic substrates. In Arabidopsis, FMOs of the YUCCA subgroup are capable of converting tryptamine to N-hydroxyl-tryptamine (Zhao et al., 2001), whereas members of the S-oxygenation subgroup (FMO_GS-OX_) oxidize the sulfide group of Met-derived methylthioalkyl glucosinolates to sulfoxide moieties, thereby generating methylsulfinylalkyl glucosinolates (Li et al., 2008). A third subgroup consists of FMO1 and a pseudogene (Olszak et al., 2006; Schlaich, 2007). Interestingly, besides the inability of *fmo1* to mediate Pip-induced resistance, *fmo1* over-accumulates Pip in the pathogen-inoculated tissue during the later stages of infection. These observations are consistent with the hypothesis that FMO1 could be involved in the oxidation of Pip or a Pip derivative in the Pip signal amplification pathway (Návarová et al., 2012).

Besides FMO1, PHYTOALEXIN-DEFICIENT4 (PAD4) and NPR1 constitute two other necessary components of both SAR and Pip-mediated resistance (Mishina and Zeier, 2006; Jing et al., 2011; Návarová et al., 2012). The lipase-like protein PAD4 is a positive regulator of SA biosynthesis and downstream signaling in plant defense (Zhou et al., 1998; Jirage et al., 1999). A similar double regulatory role exists for PAD4 also in the Pip pathway, since PAD4 not only promotes pathogen-induced Pip production but is also required for resistance promoted by Pip application (Návarová et al., 2012). PAD4 seems to exert its central defense regulatory role via transcriptional control of Pip- and SA-pathway genes, including *ALD1, FMO1*, and *ICS1* (Figure 3; Song et al., 2004a; Bartsch et al., 2006; https://www.genevestigator.com).

How do the Pip and SA defense regulatory pathways relate to each other? The *ics1* mutant accumulates Pip in a wild-type-like manner in *P. syringae*-inoculated leaves, and exogenous Pip is able to significantly increase basal resistance to *P. syringae* in *ics1*, albeit not to the same extent as in the wild-type. These findings indicate that in the pathogen-inoculated leaves, Pip increases occur independently of *ICS1*-dependent SA biosynthesis, and suggest a partial competence for Pip to induce resistance in an SA-independent manner. By contrast, Pip-induced resistance is minimal in the *npr1* mutant. Thus, a function of NPR1 in Pip signal transduction that is unrelated to its well-described SA downstream regulatory function was proposed (Návarová et al., 2012).

These partly independent traits of the Pip and SA resistance pathways diminish when the distal rather than the locally infected tissue is considered. In the distal leaves of plants that were inoculated with pathogen on other leaves, SA content increase was fully dependent on *ALD1* and hence functional Pip biosynthesis, and downstream signaling involving *FMO1* (Song et al., 2004a; Mishina and Zeier, 2006; Návarová et al., 2012). Conversely, systemic Pip accumulation strongly relies on *FMO1* and *ICS1*-mediated SA biosynthesis (Návarová et al., 2012). This reflects the afore-mentioned strong subjection of SAR establishment on effective signal amplification involving feedback mechanisms that integrate both Pip and SA signaling (Figure 3).

Above-described findings implicate a central role for the Pip resistance pathway for SAR. This is corroborated by a recent high throughput forward genetic screen for SAR-deficient Arabidopsis mutants (Jing et al., 2011). Amongst the 16 independent SAR-defective mutants identified were six *fmo1*, four *ald1*, and one *pad4* alleles, as well as three *ics1* alleles. SAR is influenced by the availability of light and depends on intact phytochrome signaling (Zeier et al., 2004; Griebel and Zeier, 2008). A more recent study suggests that the duration of light exposure after bacterial infection influences the importance of individual signals for SAR. For instance, Arabidopsis *dir1, gly1*, and *bsmt1* mutants proved SAR-defective when the SAR-inducing inoculation occurred late during the daylight period but were SAR-competent when the primary inoculation was performed early during the daylight period (Liu et al., 2011a). This suggests that the contributions of DIR1, G3P, and MeSA to SAR establishment are less prominent when plants receive a prolonged period of light after pathogen contact. The same study indicates that *FMO1* is necessary for systemic resistance induction irrespective of the light regime applied (Liu et al., 2011a), suggesting that the FMO1 pathway is a point of convergence of various SAR signals, and a critical component for SAR under varying environmental conditions (Figure 3).

#### Is Pip a SAR long-distance signal?

In *P. syringae*-inoculated leaves, Pip production occurs along with the accumulation of several other pathogen-inducible metabolites (Griebel and Zeier, 2010; Ward et al., 2010; Chanda et al., 2011; Návarová et al., 2012). In distal leaves, a more specific response occurs and the increases in a relatively small number of metabolites, including SA, SA-glucoside (SAG), and Pip occurs (Návarová et al., 2012). Návarová et al. (2012) have performed a detailed comparative analysis of the composition of Pex collected from mock-treated and virulent *P. syringae* pv *maculicola* (*Psm*)-inoculated leaves between 6 and 48 h, a time window during which the SAR long-distance information is transduced from the pathogen-inoculated to the distal leaves in their experimental system (Mishina et al., 2008). The applied methods allowed the detection and quantification of 30 defense-related metabolites and amino acids in Pex, including free SA, SAG, MeSA, AzA, JA, camalexin, and Pip. Strikingly, the only substance that exhibited a substantial (7-fold) increase in Pex from *Psm*-inoculated compared to Pex from mock-treated leaves was Pip. SA, AzA, JA, and camalexin, were not enriched in Pex collected from *Psm*-inoculated leaves, and Phenylalanine, SAG and MeSA showed only a small, 1.5- to 2-fold increase. Notably, many substances that strongly accumulated in *Psm*-inoculated leaves during the sampling period were not enriched in the respective Pex.

This selective and marked enrichment of Pip in Pex collected from *Psm*-inoculated leaves during SAR induction is consistent with the hypothesis of a Pip-specific transport out of inoculated leaves and, possibly, translocation of Pip to systemic leaves Návarová et al. (2012). Thus, a scenario is feasible in which Pip, after massive local accumulation, is transported from inoculated to distal leaves, leading to initial, moderate rises in systemic Pip levels (Figure 3). Consistent with this hypothesis, Návarová et al. (2012) detected small but significant pathogen-induced rises in distal leaves of *fmo1* which are supposed to result from transport rather than *de novo* synthesis, because *fmo1* lacks systemic up-regulation of the Pip biosynthesis gene *ALD1*. These modest systemic rises in Pip originating from transport could then drive further Pip production in the wild-type via up-regulation of *ALD1* and subsequent FMO1-mediated activation of the Pip amplification cycle, and augmented Pip in systemic leaves would then potentiate the action of other SAR long-distance signals to fully realize SAR (Figure 3). However, further experimental evidence is needed to substantiate the hypothetical function of Pip as a long-distance signal. As a water-soluble amino acid, Pip would have ideal physicochemical properties to travel via the phloem.

### Regulatory aspects of the SA pathway

#### Regulation of ICS1 expression and SA accumulation during SAR

In Arabidopsis and *Nicotiana benthamiana*, stress- and pathogen-induced SA biosynthesis proceeds via isochorismate synthase (Nawrath and Métraux, 1999; Wildermuth et al., 2001; Catinot et al., 2008). Accumulation of SA in distal leaves of locally inoculated Arabidopsis requires increased systemic expression of *ISOCHORISMATE SYNTHASE1* (*ICS1*; Attaran et al., 2009). Recent studies have provided new insight into the regulation of *ICS1* transcription. Zhang et al. (2010) identified two members of the plant-specific transcription factor family ACBP60, *SAR-DEFICIENT1* (*SARD1*) and *CALMODULIN-BINDING PROTEIN60G (CBP60g)* as SAR-relevant Arabidopsis genes. Both genes are locally and systemically up-regulated upon *P. syringae*-inoculation, and the single loss-of-function *sard1* and *cbpg60g* mutants exhibited attenuated SAR. SAR and SA accumulation in both local and systemic leaves are completely lost in a *sard1 cbpg60g* double mutant. Electrophoretic mobility shift analyses indicated that both SARD1 and CBPG60g bind to the *ICS1* promoter in a sequence-specific manner (Zhang et al., 2010). The function of CBP60g but not SARD1 is dependent on calmodulin binding, and the expression of both genes is regulated by PAD4. Moreover, expression profiling indicates that CBP60g and SARD1 affect defense responses other than SA biosynthesis, and suggests a more significant role for CBG60g and SARD1 during earlier and later stages of defense activation, respectively (Wang et al., 2011). Thus, pathogen-induced *ICS1* transcription is activated by a pair of partly redundant DNA binding proteins with different regulatory and temporal properties (Zhang et al., 2010; Wang et al., 2011).

#### Perception of SA and NPR1 regulation

Accumulating SA is sufficient to induce a subset of SA-responsive SAR genes such as the classical marker *PR1* (Sticher et al., 1997). The transcriptional co-activator NPR1 is essential for SAR and is required for the predominant part of SA downstream responses, including activation of defense gene expression (Durrant and Dong, 2004). NPR1 target genes include *PR1* and a number of genes involved in protein folding and secretion, implicating a critical role of the protein secretory pathway for SAR (Wang et al., 2005). T-DNA insertions in a subset of those genes, *LUMINAL BINDING PROTEIN (BIP2), DEFENDER AGAINST APOPTOTIC DEATH1 (DAD1)*, and *SEC61*α, reduced secretion of the PR1 protein into the apoplast and the ability of the mutant plants to enhance disease resistance in response to S-methyl-1,2,3-benzothiadiazole-7-carbothioate (BTH), a chemical that triggers a SAR-like response (Wang et al., 2005). NPR1 can reside both in the nucleus and the cytosol, and nuclear localization is required to activate *PR1* transcription (Kinkema et al., 2000). In the cytosol, disulfide bridge-connected NPR1 oligomers are converted to monomers after treatment with chemical SAR inducers. SAR induction by chemical treatment or bacterial inoculation is thought to produce a reductive redox potential in the cytosol, and *in vitro* analyses indicate that similar redox changes are sufficient to trigger NPR1 oligomer to monomer transition, presumably by reduction of disulfide bonds. Moreover, NPR1 monomer transition is associated with its nuclear localization. Thus, a model was suggested in which SA accumulation during SAR provokes redox changes driving the transition from the inactive, cytosolic NPR1 oligomer to the active, nucleus-resident NPR1 monomer (Mou et al., 2003). In addition to NPR1 oligomer/monomer transitions, other mechanisms might control the subcellular localization of NPR1. Li et al. (2012) have suggested that in tobacco, the WD40 domain containing protein TRANPARENT TESTA GLABRA2 sequesters NPR1 from the nucleus and thus represses SA/NPR1-mediated defense responses.

Yeast-two-hybrid assays suggest that, in the nucleus, Arabidopsis NPR1 can interact with TGA2, TGA5, and TGA6, three closely related members of the TGA2 subclade of bZIP transcription factors that control *PR1* expression. The triple knockout mutant *tga2 tga5 tga6* is not able to establish SAR, but also exhibits about 50-fold higher basal *PR1* expression than the wild-type, suggesting that TGA factors suppress *PR1* transcription, in addition to promoting its induction in response to SA (Zhang et al., 2003). Indeed, the *PR1* promoter contains negative regulatory elements that can be bound by TGA2, in association with NPR1, thereby controlling the inappropriate activation of *PR1* in the absence of stress (Despres et al., 2000; Zhang et al., 2003; Kesarwani et al., 2007). Consistently, *in vivo* transcription assays by Rochon et al. (2006) demonstrated that TGA2 functions as a transcriptional repressor under basal conditions. In conditions of elevated SA, TGA2 is incorporated into a transactivating complex with NPR1 that stimulates *PR1* transcription. An N-terminal BTB/POZ domain of NPR1 interacts with and negates the function of the TGA repressor (Boyle et al., 2009). Moreover, a C-terminal transacting domain of NPR1 that contains two critical cysteines (Cys^521^ and Cys^529^) in an oxidized form is necessary for the activation of *PR1* transcription (Rochon et al., 2006).

Since SA was attributed a key regulatory function in inducible plant immunity and SAR (Malamy et al., 1990; Métraux et al., 1990), a bona fide SA receptor required for SA-induced defense gene activation has remained elusive. Interestingly, when expressed in yeast, tobacco NPR1 is sensitive to SA and activates the expression of genes in a stimulus-dependent manner (Maier et al., 2011). Recently, Wu et al. (2012) have identified NPR1 as a direct SA receptor, unraveling that SA perception and subsequent transcriptional activation of defense genes are contiguous events. Using equilibrium dialysis, they determined that ^14^C-labeled SA can bind to NPR1 protein with a dissociation constant comparable to those of other plant-hormone receptor-ligand interactions. Competitive binding experiments suggested that NPR1 interacts with the defense activators SA and BTH with higher affinities than with structurally related but inactive compounds such as MeSA, 4-hydroxybenzoic acid and catechol. Further, NPR1 can coordinately bind transition metals via Cys^521^ and Cys^529^, and inductively coupled plasma-mass spectrometry analyses indicated that the protein is preferentially associated with copper. Wu et al. (2012) established that SA is bound to NPR1 via the NPR1-linked copper, presumably by the coordination of the oxygen atoms of the free carboxylate group and the phenolic hydroxyl group in *ortho* position of its aromatic ring. Further, SA binding to NPR1 causes a conformational change in the C-terminal transactivation domain that favors NPR1 oligomer disassembly and liberates the transactivation domain from an inhibitory interaction with the N-terminal BTB/POZ domain, thereby promoting nuclear localization and activation of transcription, respectively (Wu et al., 2012). According to Wu et al. (2012), SA binding, but not reducing conditions (Mou et al., 2003), induces NPR1 oligomer disassembly.

The BTB domain present in the N-terminus of NPR1 is generally found in proteins that interact with Cullin 3 (CUL3) ubiquitin E3 ligase which targets specific protein substrates for degradation by the proteasome. Cell-free degradation assays indicate that NPR1 is subject to protease-mediated degradation resulting in a continuous removal of NPR1 from the nucleus (Spoel et al., 2009). This abolishes the NPR1 coactivator activity and attenuates basal defense gene expression to prevent untimely activation of SAR. Moreover, SA treatment also promotes phosphorylation of NPR1, and thus facilitates ubiquitinylation by CUL3 ubiquitin E3 ligase and NPR1 degradation (Spoel et al., 2009). Spoel et al. (2009) further showed that this phosphorylation-mediated NPR1 turnover is necessary for SAR. Their model proposes that disposal of “exhausted” phosphorylated NPR1 from the target gene promoter allows “fresh” NPR1 to reinitiate the transcription cycle, thus allowing maximum *PR* gene transcription during SAR.

Like NPR1, its paralogues NPR3 and NPR4 contain a BTB and an ankyrin repeat protein-protein interaction domain, which are characteristic for CUL3 substrate adaptors. Fu et al. (2012) observed that *npr3 npr4* mutant plants, unlike the wild-type, lacked SA-induced NPR1 degradation, and *in vitro* pull down and co-immunoprecipitation assays indicated that both NPR3 and NPR4 interact with CUL3 ubiquitin ligase. Moreover, a yeast-two-hybrid assay established that NPR1 can interact with both NPR3 and NPR4, whereby SA promotes the NPR1-NPR3 and disrupts the NPR1-NPR4 interaction. Fu et al. (2012) also demonstrated direct binding of [^3^H]-labeled SA to NPR3 and NPR4, identifying NPR3 as a low affinity and NPR4 as a high affinity receptor for SA. In contrast to the findings of Wu et al. (2012), binding assays employed by Fu et al. (2012) did not detect a considerable binding affinity of SA to NPR1. In summary, the results of Fu et al. (2012) suggest that NPR3 and NPR4 function as adaptors of CUL3 ubiquitin E3 ligase and control NPR1 stability in an SA-dependent manner. This control mechanism seems to be required for ETI and SAR, because the *npr3 npr4* double mutant exhibited attenuated ETI and reduced HR. Fu et al. (2012) also observed that systemic resistance could not be enhanced further by prior exposure to an Avr strain of *P. syringae* in the *npr3 npr4* mutant. Hence, they concluded that the *npr3 npr4* double mutant is SAR-defective. However, results presented in Fu et al. (2012) also show that PTI associated basal resistance was significantly higher in the *npr3 npr4* double mutant than in wild-type plants. In fact, basal resistance in the *npr3 npr4* double mutant was higher than the heightened resistance observed in SAR expressing wild-type plants (Fu et al., 2012). Thus, any interpretations on SAR in the *npr3 npr4* double should take into consideration the hyper-resistant state of the *npr3 npr4* double mutant plant. Fu et al. (2012) present a model in which NPR4 binds to and promotes NPR1 degradation in the presence of low SA levels to attenuate defense gene expression under basal conditions. The model also proposes that elevated SA following SAR establishment promotes the disruption of the NPR1-NPR4 complex but is not sufficient for promoting association of the low affinity SA receptor NPR3 with NPR1, thereby liberating NPR1 to activate defense gene expression.

In addition to *NPR1*, a genetic screen has identified *Non-Recognition-of-BTH4 (NRB4)* as a mediator of SA responses in Arabidopsis (Canet et al., 2012). Plants carrying weak *nrb4* alleles exhibit strong SA insensitivity and show, to a varying degree, attenuated SAR and compromised basal resistance to *P. syringae.* Like *npr1, nrb4* mutants fail to develop SA- or BTH-induced resistance and over-accumulate SA in the course of *P. syringae*-infection. *nrb4* null alleles also express severe growth defects, indicating a role of *NRB4* in plant development. *NRB4* is allelic to Mediator subunit 15 (*MED15*). Mediator represents a multiprotein complex that functions as a transcriptional co-activator or co-repressor in eukaryotes, depending on the nature of associated protein components. Individual Mediator subunits transduce diverse signals to the general transcriptional machinery and can thereby convey plant transcriptional responses to specific stimuli (Kidd et al., 2011). An Arabidopsis screen for reduced *PR1* activation upon exogenous NAD^+^ application, a treatment that induces *PR* gene expression and disease resistance in Arabidopsis (Zhang and Mou, 2009), identified Mediator subunit 16 (MED16) as an essential SAR component (Zhang et al., 2012). M*ed16* knockout lines exhibit increased susceptibility to Avr and virulent *P. syringae* and are unable to establish SAR. Following bacterial inoculation, *med16* plants locally and systemically accumulate SA to similar levels than the wild-type but are impaired in *PR* gene expression. Zhang et al. (2012) demonstrated that *MED16* functions downstream of SA and positively regulates NPR1 protein accumulation. Beyond its function in the SA pathway, *MED15* is also required for plant defense toward necrotrophic pathogens and activation of jasmonic acid (JA)/ethylene (ET) pathway genes. Thus, MED16 seems to relay signals from the SA pathway and the JA/ET pathway to the general transcription machinery. MED16 might regulate SA responsiveness via the modulation of NPR1 protein accumulation, but it is not clear yet whether NPR1 or TGA factors are physically associated with the Mediator subunit (Zhang et al., 2012).

## SAR—an alarmed state of plants that confers defense priming via Pip accumulation

Several PR proteins exhibit antimicrobial activities *in vitro* and overexpression studies indicate that increased expression of single *PR* genes can render plants more resistant to particular pathogen types (Sticher et al., 1997). This suggests that PR proteins that accumulate during SAR contribute to increased pathogen resistance by directly exerting harmful effects to microbial invaders. A second phenomenon supposed to confer resistance during SAR is defense priming or conditioning (Conrath, 2011). Defense priming can be interpreted as an alarmed or sensitized state of plants during which they are able to react more quickly and effectively to pathogen attack.

Although plant conditioning has been associated for a long time with biologically induced SAR (reviewed in Sticher et al., 1997), the phenomenon has been most convincingly described for experimental setups in which plants or plant cell cultures were exogenously treated with chemical enhancers of resistance. These compounds include plant-derived substances such as SA, thiamine and riboflavin (Thulke and Conrath, 1998; Ahn et al., 2007; Zhang et al., 2009), but often also synthetic or unnatural substances like BTH or β-amino butyric acid (BABA; Katz et al., 1998; Zimmerli et al., 2000). Recently, a high-throughput chemical screen identified a series of novel synthetic compounds that confer defense priming by targeting SA glycosyltransferases and thus increasing endogenous SA accumulation (Noutoshi et al., 2012).

Recent studies indicate that a primary inoculation with a SAR-inducing pathogen leads to defense priming in distal leaves, enabling the whole plant to more effectively mobilize defenses in the course of a subsequent challenge infection (Jung et al., 2009; Návarová et al., 2012). Jung et al. (2009) demonstrated that biological SAR induction, similar to exogenous AzA treatment [see section “Azelaic Acid (AzA)”], enables plants to accumulate higher levels of SA and *PR1* transcripts. This effect was not observed in plants disrupted for the *AZI1* gene, which is transiently expressed at elevated levels in response to AzA treatment (Jung et al., 2009). However, genetic evidence that AzA is responsible for priming of SA production and responsiveness during biological SAR is lacking. Beckers et al. (2009) reported enhanced activation of mitogen-activated protein kinases (MPK) MPK3 and MPK6 upon mechanical stress (pressure infiltration of water) or *P. syringae*-exposure of leaves when Arabidopsis plants were previously treated with BTH. They found that full BTH-mediated priming of *PAL1*- and *PR1*- expression in response to mechanical stress was dependent on both *MPK3* and *MPK6.* MPK3 but not MPK6 was also required for *P. syringae*-induced SAR. However, the role of the MPKs in priming of SAR-related defense responses to pathogen challenge following biological SAR induction was not investigated (Beckers et al., 2009). Another study established that BTH application and localized *P. syringae*-treatment systemically primed Arabidopsis for enhanced expression of the WRKY transcription factor genes *WRK6, WRKY29*, and *WRKY53* in response to the stress associated with pressure-infiltration of water into leaves (Jaskiewicz et al., 2011). This priming of *WRKY* genes by BTH was dependent on *NPR1*. Concomitantly, BTH-treatment and *P. syringae*–inoculation also induced histone modifications in the chromatin at the promoters of these *WRKY* genes, suggesting that these histone modifications provide a form of memory of a previous stress. However, whether this histone modification-associated memory has a role in SAR-mediated priming and establishment of systemic immunity remains to be determined.

The recent study of Návarová et al. (2012) demonstrated that biologically-induced SAR in Arabidopsis plants promotes an alarmed state that accelerates the responses to subsequent pathogen attack on several levels. On the metabolite level, SAR priming is characterized by a strongly potentiated induction of both Pip biosynthesis and accumulation of the phytoalexin camalexin after *P. syringae* inoculation, and by a more moderate stimulation of SA accumulation. Moreover, biological SAR prepares plants for a stronger induction of defense genes after a challenge infection, including the two essential SAR regulatory genes *ALD1* and *FMO1*, and the SA-inducible *PR1*. The Pip-deficient *ald1* plants are defective in these SAR-associated conditioning events, suggesting that Pip accumulation is critical for SAR priming. This is corroborated by the findings that exogenous Pip promotes a sensitized state highly similar to that occurring after biological induction of SAR and compensates priming defects in *ald1*. Therefore, genetic and physiological evidence indicates that Pip accumulation is necessary and sufficient to promote a primed state after biological SAR induction (Návarová et al., 2012). Interestingly, the biosynthesis of the endogenous priming regulator Pip is also potentiated during biological SAR, indicating that feedback amplification mechanisms similar to those described in section “The Pip Resistance Pathway Is Central for SAR” for SAR establishment contribute to defense priming in the course of the challenge infection. Moreover, the observations that Pip also accumulates in BABA-treated plants to physiological levels, and that Pip-deficient *ald1* plants are defective in BABA-induced resistance to *P. syringae* suggest that BABA-induced resistance to hemibiotrophic bacteria is regulated via Pip-mediated priming events (Návarová et al., 2012).

## The memory of SAR is passed on to the progeny

SAR confers a fitness advantage under conditions of disease stress (Traw et al., 2007). A recent study indicated that the memory of SAR in Arabidopsis is passed on to the next generation, thus benefiting the progeny plants as well (Luna et al., 2012). The progeny of plants in which SAR had been activated by inoculation with a virulent strain of *P. syringae* pv *tomato* exhibited heightened resistance to *P. syringae* pv *tomato* as well as the unrelated oomycete *H. parasitica* than the progeny of plants that received a control mock-treatment. Although the basal content of defense hormones SA, JA, and JA-Ile were not altered in these next generation SAR plants, SAR associated defenses were more responsive to SA, as indicated by the more robust expression of *PR1* and the *WRKY* genes, *WRKY6, WRKY53* and *WRKY70* in these progeny when treated with SA, than in progeny of plants in which SAR was not induced (Luna et al., 2012). NPR1 was required for the next generation SAR. By contrast, the sensitivity of these next generation SAR progeny to JA was reduced, resulting in the weaker induction of JA-inducible genes (*PDF1.2* and *VSP2*) in response to exogenously applied JA and a concomitant increase in susceptibility to the necrotrophic pathogen *Botrytis cinerea*. Similarly, enhanced protection in progeny plants has also been reported for plants treated with an Avr strain of *P. syringae* or BABA (Slaughter et al., 2012). Progeny of the BABA-treated plants were primed for SA-dependent resistance against *P. syringae* and *H. arabidopsidis*.

Luna et al. (2012) showed that next generation SAR was accompanied by changes in the methylation and acetylation status of histones at the promoters of various *NPR1* regulated or SAR associated genes, including *PR1, WRKY6*, and *WRKY53*. Promoters of these genes in plants exhibiting next generation SAR contained elevated levels of histone 3 with acetylated Lys9 (H3K9ac), which is considered a transcription activation mark. By contrast, the *PDF1.2* promoter contained elevated levels of H3K27me3, which is normally associated with transcriptional silencing. These results suggest that plants exhibiting next generation SAR have chromatin marks that likely are involved in retaining memory of an infection in the parental generation. In the absence of any evidence that histone modifications *per se* can be transmitted via the gametes, Luna et al. (2012) suggested that DNA methylation patterns, which can be transferred from one generation to another, are likely connected with transmission of memory associated with SAR from the parental generation to the progeny. Bacterial infection is known to cause hypomethylation (Pavet et al., 2006). Similarly, JA and SA treatment also have been reported to impact the DNA methylation status (Verhoeven et al., 2010). Luna et al. (2012) noted that basal resistance was higher in the *drm1 drm2 cmt3* triple mutant in which non-CpG DNA methylations are reduced. In addition, the *drm1 drm2 cmt3* plants also responded more robustly to SA thus mimicking the priming effect associated with next generation SAR. However, Slaughter et al. (2012) did not see any relationship between next generation protection conferred by BABA or bacterial inoculation and the methylation status at the *PR1* promoter, thus suggesting that if DNA methylation changes are associated with transmission of the priming memory from the parent to the progeny, it is exerted not directly at the *PR1* promoter, but rather at the level of upstream regulatory genes. Next generation stress protection is not limited to defense against pathogens. It has also been reported in Arabidopsis and tomato (*Solanum lycopersicum*) subjected to mechanical damage or herbivory (Rasmann et al., 2012). In this case, the next generation protection was accompanied by priming of JA-dependent defenses. Epigenetic changes associated with next generation protection offer the advantage that they are not permanent and hence offer plasticity, which allows plants to better adapt to a changing environment.

## Concluding remarks

Although SAR confers a fitness advantage that can benefit multiple generations of plants (Traw et al., 2007; Luna et al., 2012), it needs to be tightly regulated since it is an energy-driven process that diverts resources from growth and development (Heidel et al., 2004; Pajerowska-Mukhtar et al., 2012). Hence, uncontrolled and untimely activation of SAR is detrimental for plant growth and development. Pathogens are also known to target plant defenses to facilitate infection. A circuitry involving networking between multiple signals (Figure 3) offers plants the advantage of having sufficient flexibility to better control SAR under different environmental conditions. The coming years will be important for understanding the molecular components of this circuitry, its regulation, conservation amongst plants and the application of this knowledge to sustainable agriculture.

### Conflict of interest statement

The authors declare that the research was conducted in the absence of any commercial or financial relationships that could be construed as a potential conflict of interest.
